# Supplementary material for: Deglacial variability of South China hydroclimate heavily contributed by autumn rainfall
Source: Nat Commun. 2021 Oct 7;12:5875. doi: 10.1038/s41467-021-26106-0 (PMC8497617; doi:10.1038/s41467-021-26106-0)
Supplement: Supplementary file 1 — supplemental material [file 41467_2021_26106_MOESM1_ESM.pdf]

Supplementary Information

Deglacial variability of South China hydroclimate heavily contributed by autumn rainfall

Chengfei He[1,2,3*], Zhengyu Liu[2], Bette L. Otto-Bliesner[4], Esther C. Brady[4], Chenyu Zhu[5,3], Robert Tomas[4], Sifan Gu[6,3], Jing Han[7], Yishuai Jin[5]

1. College of Atmospheric Sciences, Nanjing University of Information Science and Technology, Nanjing, China
2. Department of Geography, The Ohio State University, Columbus, OH, USA
3. Open Studio for Ocean-Climate-Isotope Modeling, Pilot National Laboratory for Marine Science and Technology, Qingdao, China
4. Climate and Global Dynamics Laboratory, National Center for Atmospheric Research, Boulder, CO, USA
5. Key Laboratory of Physical Oceanography, Ocean University of China, Qingdao, China
6. School of Oceanography, Shanghai Jiao Tong University, Shanghai, China
7. Department of Atmospheric and Oceanic Sciences, Peking University, Beijing, China

Nature Communications

Mar 22nd, 2021, submitted

Jul 20th, 2021, 1st revision

Aug 28th, 2021, 2nd revision

Corresponding Author, Email: Chengfei He, he.1519@osu.edu

SON precipitation in ice and KMT run

The SON precipitation in our ice and KMT (ocean bathymetry) run is almost constant at South China, but changes abruptly at 14ka and 12ka, when we changed the KMT. To separate the role of continent ice sheet and KMT, an independent KMT simulation that keeps ice sheet unchanged, was branched at 14ka, and lasts for 500 years. The last 100 years' data was examined. It turns out that the abrupt change is entirely due to the KMT (cyan dots in Supplementary Fig.2b).

We speculate that the evolution of real world precipitation in response to KMT would be much smoother, following the sea level rise, instead of an abrupt change as in our model. To avoid the "artificial jump", we removed the responses of KMT and ice sheet in circulation and precipitation before MCA analysis in present study, and took HS1 and LGM as example to illustrate the mechanism. We caveat that the KMT and ice sheet effect maybe larger in YD, as the sea level has risen a lot. In addition to autumn monsoon, the summer monsoon is also strongly influenced by the KMT change, in particular at 14ka (Supplementary Fig.2a). More study will be conducted to reveal the relation between KMT and East Asian monsoon.

Summer monsoon rainfalls in cold stadials of HS1 and YD

The rainfall response in summer monsoon shows a two-phased change in East China during HS1. In the early summer, the hydroclimate in East China is controlled more by the cooling due to the AMOC, as the anomalous Meiyu rainfall belt is still trapped in South China due to the reduced southerly monsoon flow, southward displacement of the westerly jet[1–3], and the associated silk-road teleconnection[3] (Supplementary Fig. 1e and Supplementary Fig. 2a). However, in mid-summer, the insolation induced warming overwhelms the cooling in the

Northern Hemisphere in HS1, which leads to a deepening of the Subtropical High, an intensified southerly wind in East China and in turn a wet North China (Supplementary Fig. 1e and Supplementary Fig. 3b). The mid-summer response is slightly dominant, leading to a weak rainfall increase in the summer season in HS1 relative to LGM (supplementary Fig.2d) and As both the South and North China become wet in HS1 relative to LGM. In contrast, the rainfall in East China shows a clear dipole pattern from BA to YD — wet in the south and dry in the north (Supplementary Fig. 2c), consistent with available observations[4,5] (also see Fig 2 in He et al.[3]). From the perspective of single forcing effect, both the orbital forcing and meltwater flux force a dipole response in the summer monsoon between North and South China (red, and blue between Supplementary Fig. 2a and d), with the latter response substantially weaker in North China than in South China. From LGM to HS1, the longer duration (~5000 years) leads to a strong insolation forced rainfall increase in North China that overwhelms the weak rainfall reduction response to meltwater forcing and lead to a wet North China. From BA to YD, however, the insolation forcing change is weak due to the short duration (~1000 years), therefore the meltwater forced dipole response becomes dominant. In South China, we also note that the weakening trend of rainfall forced by the insolation forcing is partly canceled by the increasing trend forced by rising GHG (Supplementary Fig.2a), leaving the response to meltwater flux as the dominant millennial response throughout the deglaciation.

Finally, the East Asian summer monsoon rainfall and water isotope evolution in the last deglaciation is discussed in more detail in He et al.[3]

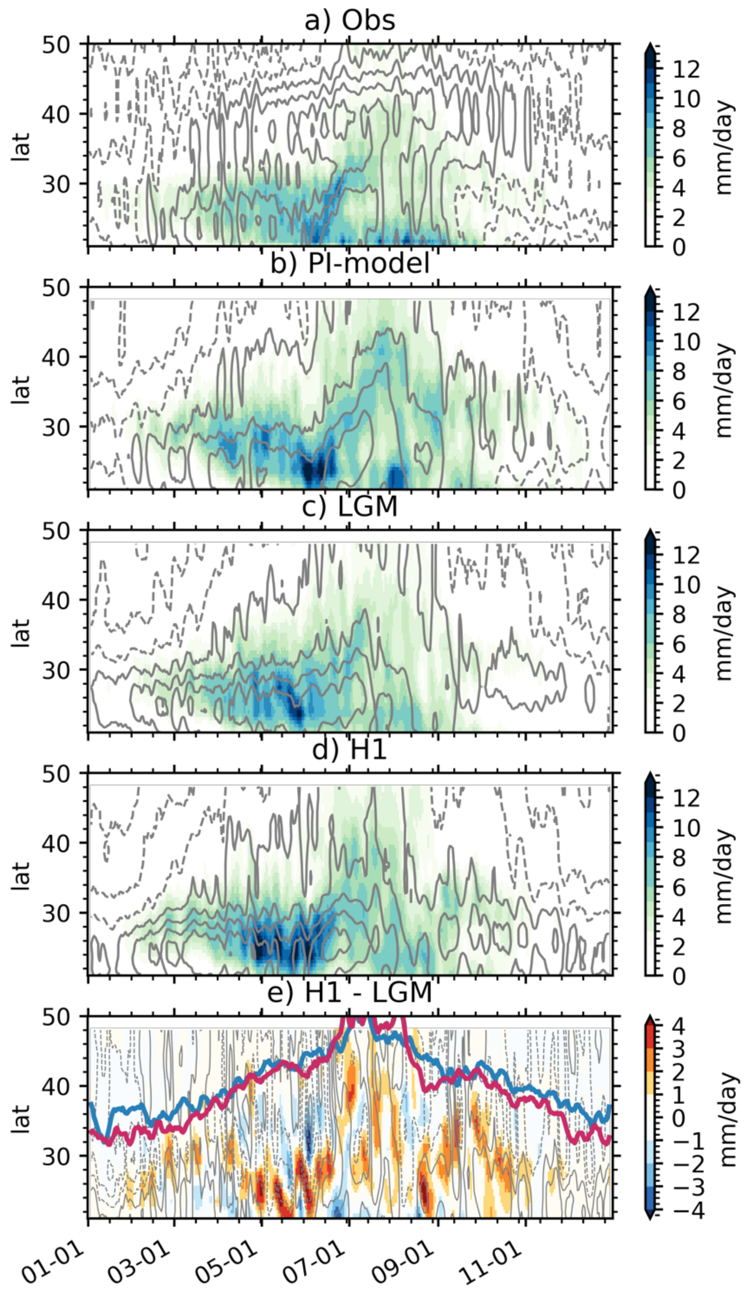

Supplementary Fig. 1. **Hovmöller diagram of the precipitation and monsoon flow climatology in East China.** (a) Observation (shading) from APHRODITE dataset[6] and meridional velocity in1000-850hPa from NCEP/NCAR reanalysis-1[7]. (b), (c), and (d) as in (a) bur for model at present day, LGM, HS1, respectively. (e) difference between HS1 and LGM (HS1 - LGM). In (a), observation data spans from 1951 to 2007. In (e), the blue and red curves corresponds to the westerly jet location at LGM and HS1, respectively. The location is defined as statistical expectation of where the maximum zonal wind locates in 400-200hPa, determined by the 6 hourly output from the iCAM snapshot simulations. In (a) and (e), the contour interval is 1 m/s, while others are 2 m/s. Solid contour denotes southerly wind, and dashed contour denotes northerly wind.

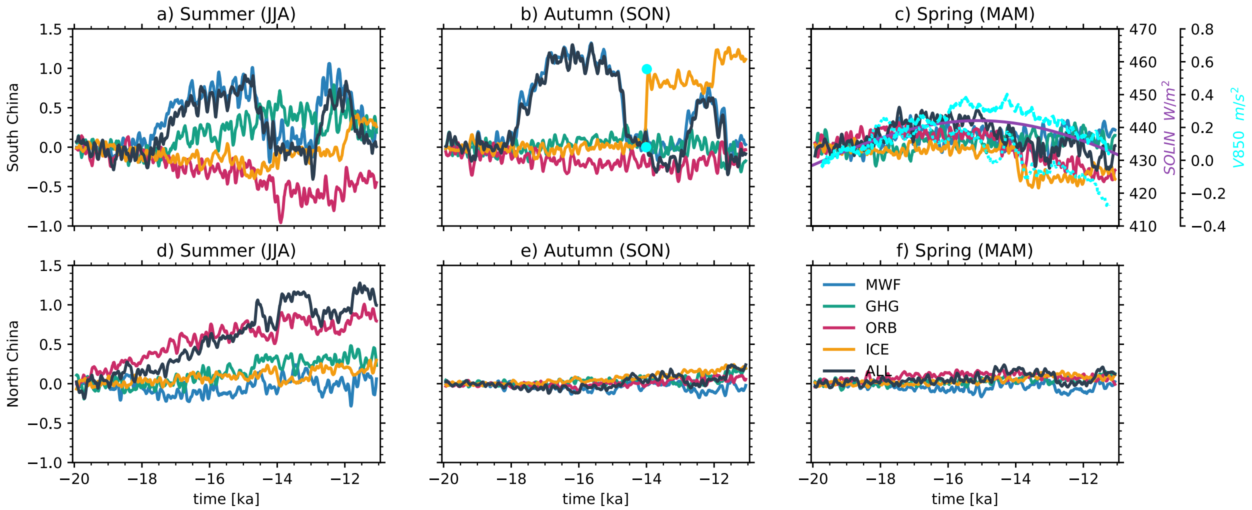

Supplementary Fig. 2. **Summer and Autumn precipitation time series in response to different climate forcings.** (a) Summer precipitation for South China [20-35°N, 108-120°E] in response to single climate forcing; (b, c) as in (a), but for Autumn and Spring precipitation; (d) as in (a), but for North China [37-50°N, 108-120°E]; (e, f) as in (d), but for Autumn and Spring precipitation. In (b), the precipitation in ice run abruptly changes at 14ka and 12ka, which is entirely due to the change of KMT (cyan dots, see supplementary material). In (a)-(f), the climatology of LGM is removed; MWF = ICE + ORB + GHG + MWF – ICE + ORB + GHG; GHG = ICE + ORB + GHG – ICE + ORB; ORB = ICE + ORB – ICE; ICE = ICE – LGM climatology, see Methods for different runs. In (c), the local MAM solar insolation is plotted in purple, and the monsoon flow V850 is plotted in cyan, with dashed as effect due to orbital forcing and dotted as effect from all-forcing. Curves are smoothed by a 9-decadal running mean for presentation and by a 50-decadal running mean for V850.

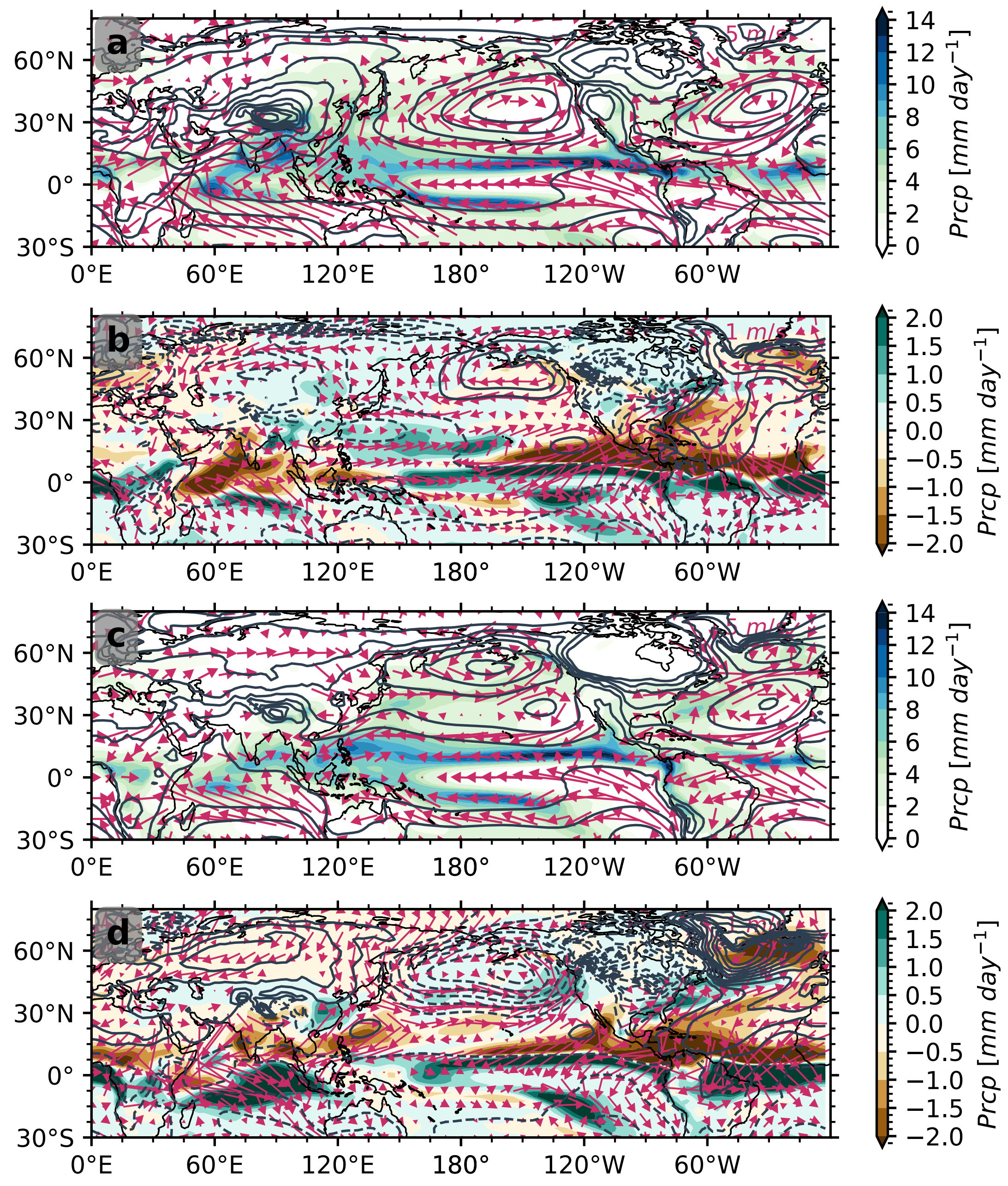

Supplementary Fig. 3. **Summer and Autumn climate at LGM and their responses in HS1 (HS1 - LGM).** (a), summer precipitation (shading), sea level pressure (contour) and low-level atmospheric circulation (vector) at LGM. (b), as in (a) but for summer anomaly.(c), (d) as in (a), (b) bur for autumn. In (a) and (c), the contours span from 950 to 1050 hPa, with interval as 5 hPa. In (b) and (d), the contours span from -10 to 10 hPa, with interval as 1 hPa. The low-level atmospheric circulation is defined as averaged wind in 850 to 1000 hPa.

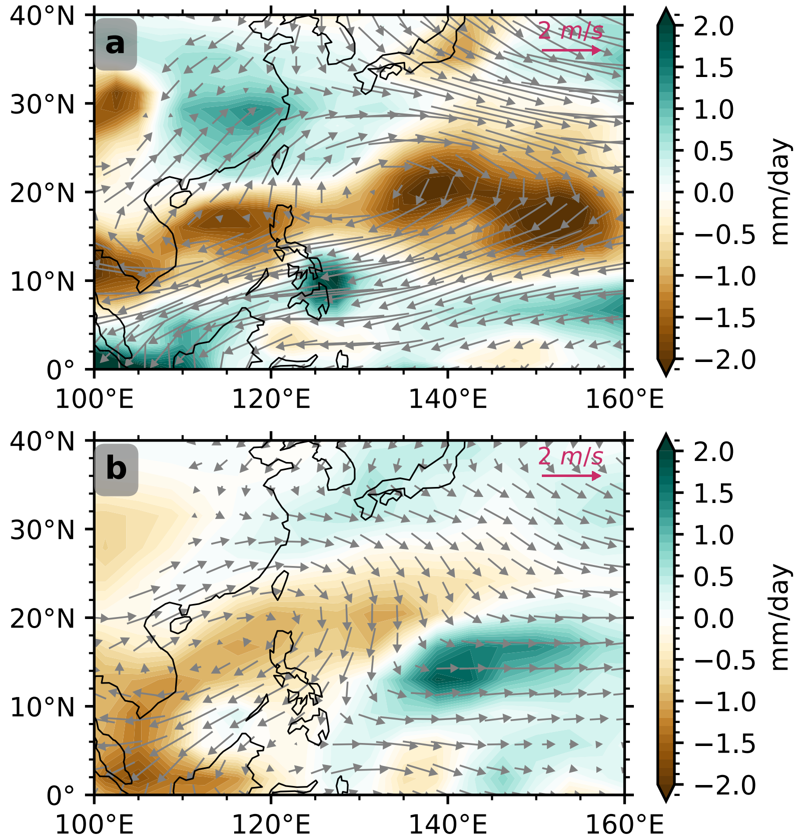

Supplementary Fig. 4. **Autumn climate response between HS1 and LGM (HS1 - LGM).** (a) iCAM. (b) TRACE21ka. Shading: precipitation; Vector: 850 hPa wind.

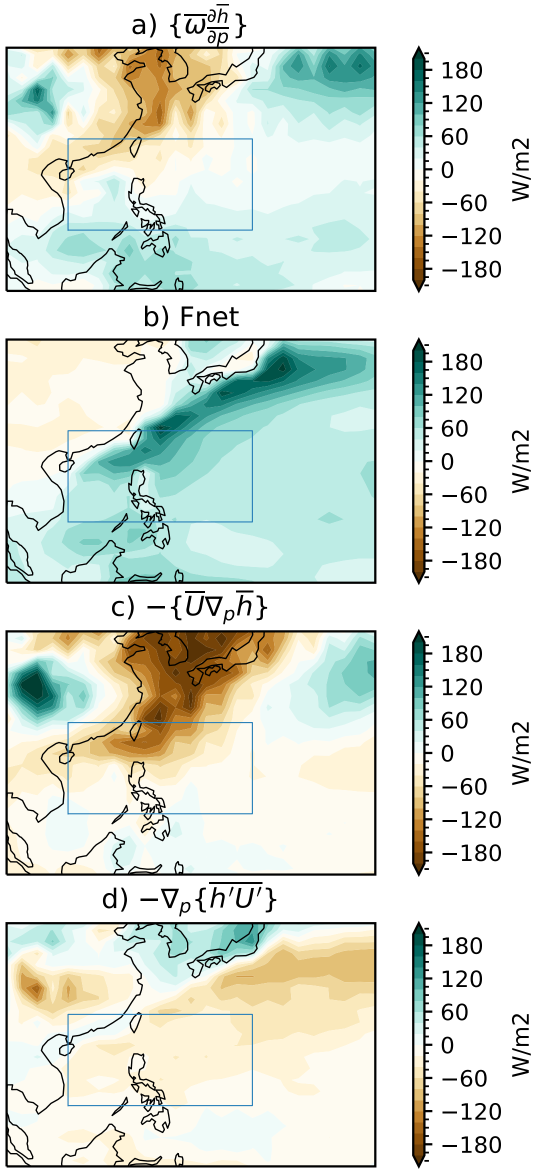

Supplementary Fig. 5. **Autumn climatology of the MSE budget terms at LGM.** (a) vertical MSE advection; (b) net energy source; (c) horizontal MSE advection; (d) transient eddy MSE flux. In (a), positive sign denotes MSE export, and vice versa. In (b) to (d), positive sign denotes MSE import, and vice versa. See Methods for detailed discussion of each term.

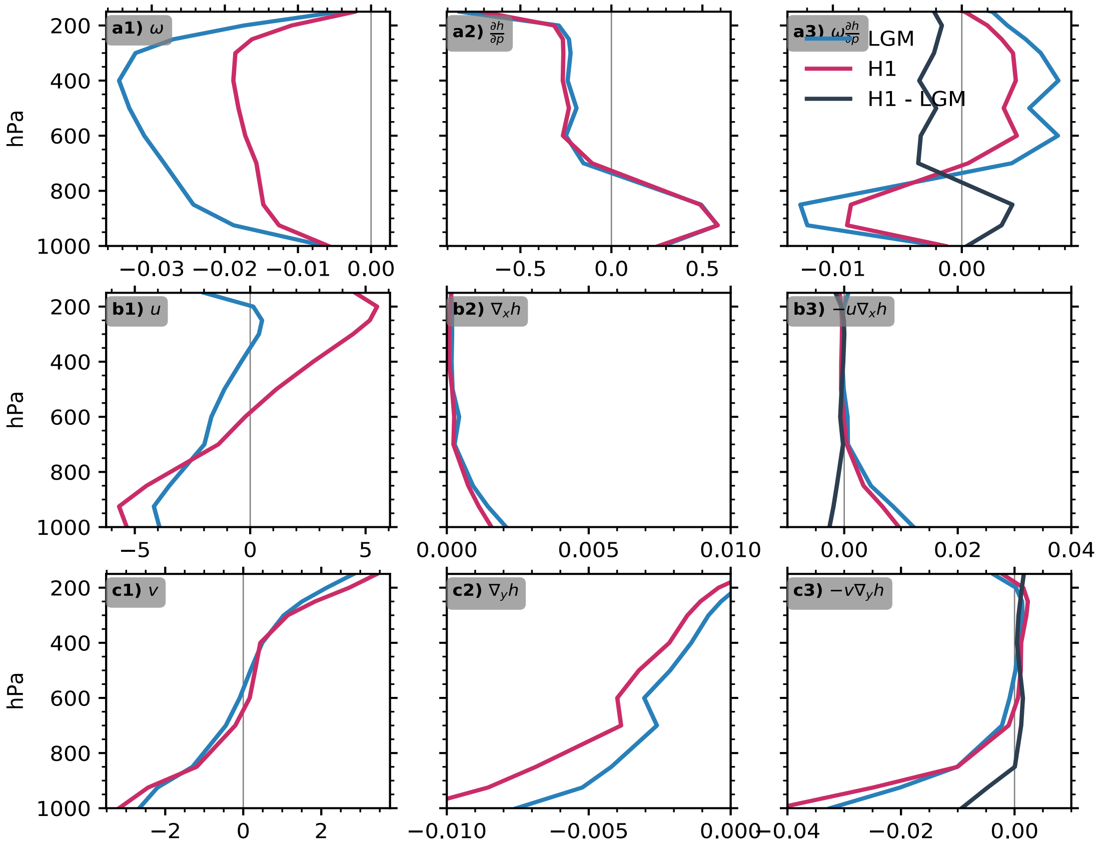

Supplementary Fig. 6. **Vertical profile of the region averaged variables associated with MSE budget terms at LGM and HS1 in [10-25ºN, 110-140ºE].** (a1) Vertical p-velocity (Pa/s); (a2) vertical MSE gradient (J/kg/Pa); (a3) vertical MSE advection (J/kg/s); (b1)-(b3), and (c1-c3) as in (a1)-(a3) bur for zonal and meridional MSE advection, unit in m/s, J/kg/m, and (J/kg/s). See legend in (a3) for different curves.

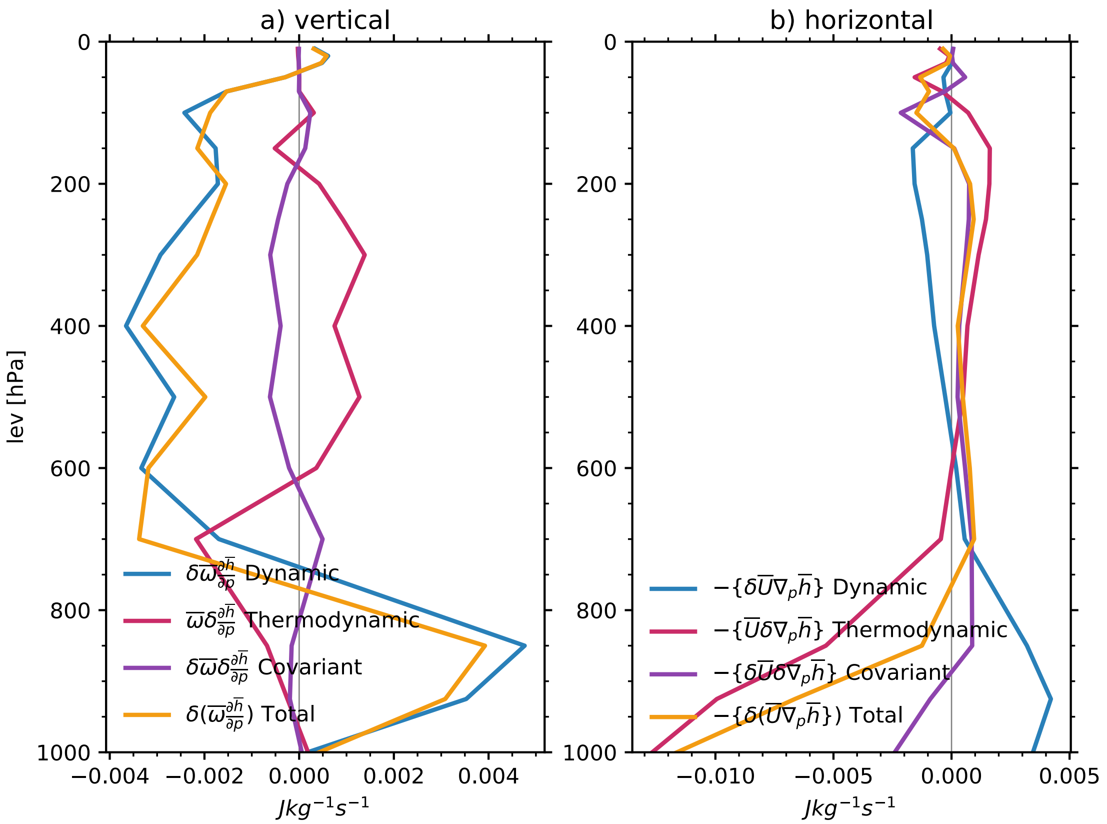

Supplementary Fig. 7. **Vertical profile of decomposition of vertical and horizontal MSE advection responses.** (a) Vertical MSE advection anomaly (yellow) is portioned into dynamic part associated with vertical motion change (blue), thermodynamic part associated with MSE gradient change (red), and the covariant part (purple). (b) as in (a), but for horizontal MSE advection anomaly. See Methods for details.

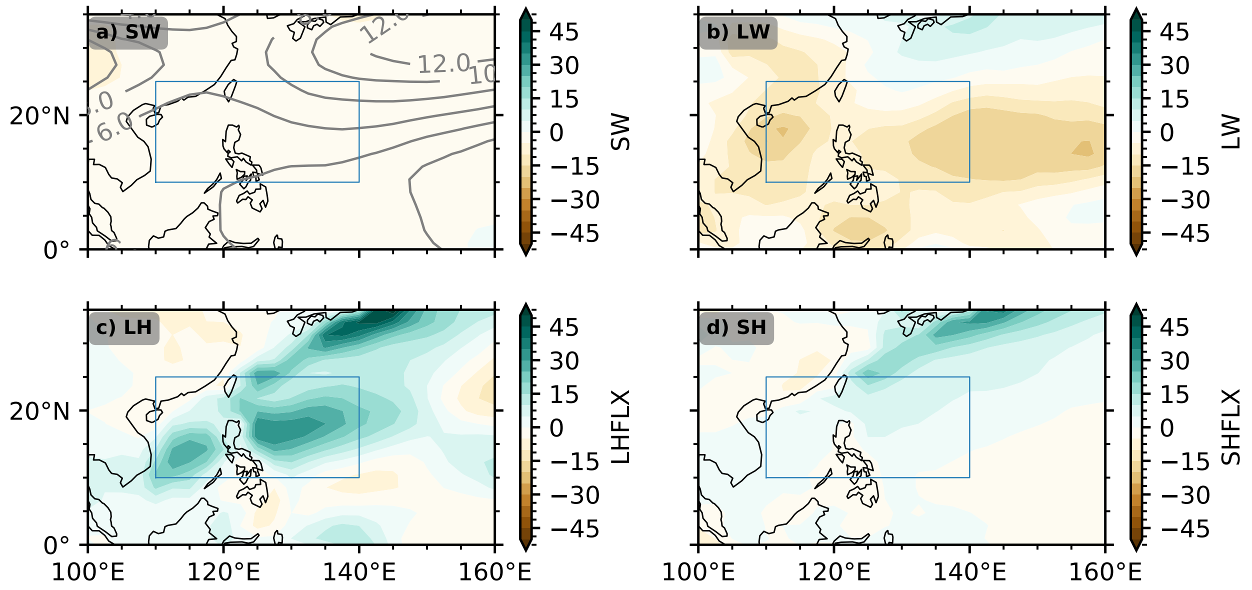

Supplementary Fig. 8. **Heat flux responses between HS1 and LGM (HS1 - LGM) in SON.** (a) Shortwave radiation (shading) and zonal wind velocity at 200 hPa (contour, m/s); (b) Longwave radiation; (c) Latent heat flux; (d) Sensible heat flux. Positive sign denotes energy import to atmosphere and vice versa. The box is where WNPAC is located, as shown in Fig. 3. Unit: W/m^2.

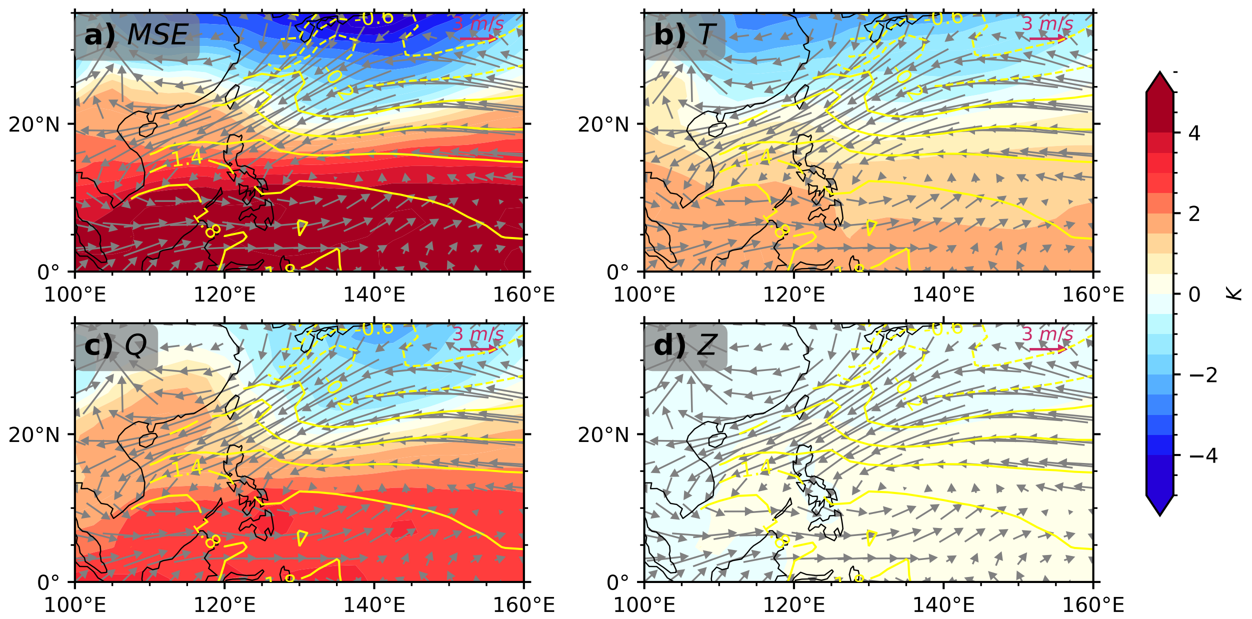

Supplementary Fig. 9. **Autumn low-level (850-1000 hPa) MSE responses and its components between HS1 and LGM (HS1 - LGM).** (a) total MSE; (b) temperature; (c) water vapor; (d) geopotential height. Overlaid is the climatology SON 850 hPa wind at LGM. In (a)-(d), the yellow contour denotes SST anomaly, with solid as positive and dash as negative, and contour interval is 0.4 ^{o}C. The unit of MSE and its components are converted into Kelvin (K) for presentation.

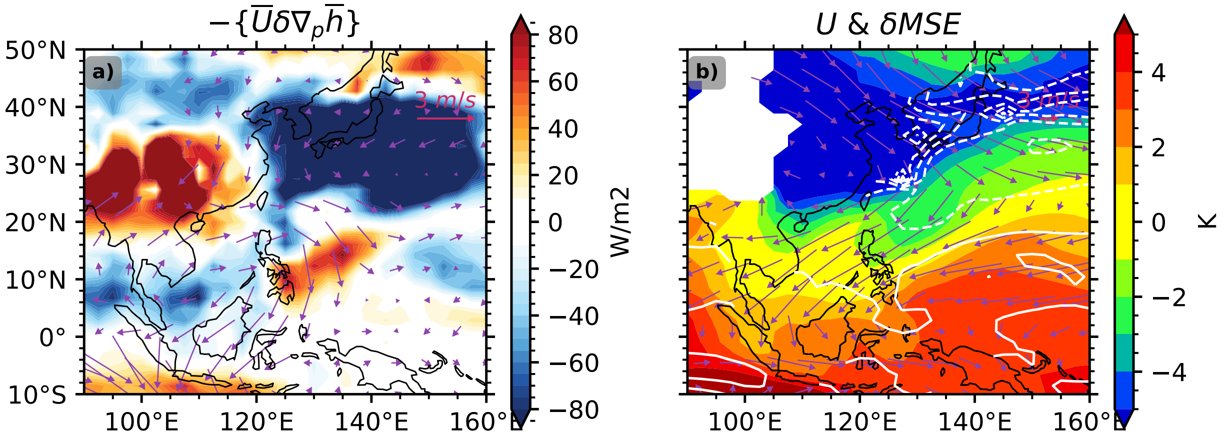

Supplementary Fig. 10. **WNPAC response (HS1 - LGM) in winter.** (a) anomalous MSE advection associated with MSE gradient change (shading) and circulation anomaly (vector); (b) anomalous low-level (850-1000hPa) atmosphere MSE (shading), mean winter circulation (vector) and SST anomaly (contour). In (b), the contours span from -3 to 3ºC, with interval as 0.5 ºC.

References

1. Liu, Z. et al. Chinese cave records and the east asia summer monsoon. *Quaternary Science Reviews* **83**, 115-128 (2014).
2. Zhang, H. et al. East Asian hydroclimate modulated by the position of the westerlies during Termination I. *Science* **362**, 580-583 (2018).
3. He, C. et al. Hydroclimate footprint of pan-Asian monsoon water isotope during the last deglaciation. *Science Advances* **7**, eabe2611 (2021).
4. Goldsmith, Y. et al. Northward extent of East Asian monsoon covaries with intensity on orbital and millennial timescales. *Proceedings of the National Academy of Sciences* **114**, 1817-1821 (2017).
5. Chen, F. et al. East Asian summer monsoon precipitation variability since the last deglaciation. *Scientific Reports* **5**, 1-11 (2015).
6. Yatagai, A. et al. APHRODITE: Constructing a Long-Term Daily Gridded Precipitation Dataset for Asia Based on a Dense Network of Rain Gauges. *Bulletin of the American Meteorological Society* **93**, 1401-1415 (2012).
7. Kalnay, E. et al. The NCEP/NCAR 40-Year Reanalysis Project. *Bulletin of the American Meteorological Society* **77**, 437-471 (1996).
